# Lack of Effects of the Presence of a Dog on Pain Perception in Healthy Participants—A Randomized Controlled Trial

**DOI:** 10.3389/fpain.2021.714469

**Published:** 2021-11-05

**Authors:** Cora Wagner, Jens Gaab, Cosima Locher, Karin Hediger

**Affiliations:** ^1^Division of Clinical Psychology and Psychotherapy, Faculty of Psychology, University of Basel, Basel, Switzerland; ^2^School of Psychology, University of Plymouth, Plymouth, United Kingdom; ^3^Department of Anesthesiology, Critical Care and Pain Medicine, Harvard Medical School, Boston Children's Hospital, Boston, MA, United States; ^4^Clinic for Neurorehabilitation and Paraplegiology, REHAB Basel, Basel, Switzerland; ^5^Human and Animal Health Unit, Department of Epidemiology and Public Health, Swiss Tropical and Public Health Institute, Basel, Switzerland; ^6^Faculty of Psychology, Open University, Heerlen, Netherlands

**Keywords:** pain, animal-assisted intervention, expectation, treatment rationale, placebo, social support

## Abstract

Animal-assisted interventions (AAIs) have been shown to be effective in the treatment of pain. Studies suggest that relationships with animals can have comparable qualities to relationships with humans and that this enables animals to provide social support. Further, the presence of an animal can strengthen the therapeutic alliance between patients and treatment providers. This suggests that the analgesic effects of AAI might be mediated by social support from an animal or by strengthening the alliance between the patient and the treatment provider. To test these assumptions, we examined the effects of the presence of a dog on experimentally induced pain in a pain assessment and a pain therapy context. Hundred thirty-two healthy participants were randomly assigned to the conditions “pain,” “pain + dog,” “pain + placebo,” or “pain + placebo + dog.” We collected baseline and posttreatment measurements of heat-pain tolerance and the heat-pain threshold and of the corresponding subjective ratings of heat-pain intensity and unpleasantness as well as of participants' perceptions of the study investigator. The primary outcome was heat-pain tolerance. The presence of the dog did not influence the primary outcome (“pain” vs. “pain + dog”: difference = 0.04, CI = −0.66 to 0.74, *p* = 0.905; “pain + placebo” vs. “pain + placebo + dog”: difference = 0.43, CI = −0.02 to 0.88, *p* = 0.059). Participants did also not perceive the study investigator to be more trustworthy in the presence of the dog (“pain” vs. “pain + dog”: difference = 0.10, CI = −0.67 to 0.87, *p* = 0.796; “pain + placebo” vs. “pain + placebo + dog”: difference = 0.11, CI = −0.43 to 0.64, *p* = 0.695). The results indicate that the mere presence of a dog does not contribute to pain reduction and that the analgesic effects of AAI that previous studies have found is not replicated in our study as AAI did not increase perceived social support and had no effect on the alliance between the participant and the treatment provider. We assume that the animal most likely needs to be an integrated and plausible part of the treatment rationale so that participants are able to form a treatment-response expectation toward AAI.

**Clinical Trial Registration:** This study was preregistered as a clinical trial on www.clinicaltrials.gov (Identifier: NCT0389814).

## Introduction

Animal-assisted interventions (AAIs) are “goal-oriented and structured interventions that intentionally incorporate animals in health, education and human service for the purpose of therapeutic gains in humans” (1). AAIs have a wide range of clinically relevant effects, such as lowering symptoms in patients with depressive and anxiety disorders (2–7), improving neurohormone levels in adult patients diagnosed with advanced heart failure (8), and reducing cortisol levels in adult healthcare professionals as well as in children with insecure attachment (9, 10). Moreover, a recent meta-analysis has suggested that AAI can be an effective therapy for relieving pain in patients across all age groups (7). For example, children exhibited a significant reduction in pain perception and experience after an AAI compared to a control intervention without an animal present both in an acute pediatric setting (11) and after surgery (12). Similar effects have been reported in AAI studies on pain syndromes in adults. Patients who had 15-min visits with a therapy dog before receiving standard postoperative treatment had significantly lower perceptions of pain after total joint arthroplasty than patients who only received standard postoperative treatment (13). Adult patients with chronic pain perceived significantly less pain when they spent their waiting time with a therapy dog compared to patients in a waiting room without a dog present (14). Further, patients with fibromyalgia showed a greater decrease in pain when they were in a group that received a 20-min session with a therapy dog and its handler compared to a group that received the session with only the handler (15). However, not all studies found that AAI leads to pain reduction (16, 17). Further, previous studies differed with regard to the study design and also showed methodological weakness, such as lack of no randomization or insufficient control groups (7). Thus, the evidence base for the effects of AAI on pain is still weak, and high-quality studies are warranted to investigate the effects and the mechanisms by which AAI leads to pain reduction (7).

Although these results are promising, the mechanisms by which AAI leads to pain relief are yet to be fully understood, since it is still unclear how animals contribute to pain relief (7). Research on social support can suggest possible explanations. The mere presence of another person has been shown to lead to a reduction of perceived pain (18). This effect on pain can be found in both active (19, 20) and passive forms of social support (18), and it does not seem to depend on the degree of the relationship, that is, on whether the person is a partner, friend, or stranger (18, 21). Previous research has highlighted that relationships with animals can have comparable qualities to relationship with humans (22, 23) and that pets can provide social support for their owners (24). Furthermore, the presence of an animal can also positively influence how we perceive others and strengthen the therapeutic alliance between the patient and the treatment provider (25–27). This is of relevance since the therapeutic alliance is an important determinant of treatment outcomes in medical interventions (28), psychotherapy (29), and placebo interventions (30, 31).

The analgesic effects of AAI could thus be mediated by providing direct social support for the patient or by strengthening the alliance between the patient and the treatment provider. To test these assumptions, we examined the effects of AAI with a dog on experimentally induced pain in healthy participants, mimicking two different clinical settings: pain assessment and pain therapy. We hypothesized that participants would show increased heat-pain tolerance in both settings when a dog is present based on the assumption that the mere presence of a dog can act as direct social support. We also hypothesized that participants would show increased heat-pain threshold and decreased subjective ratings of pain intensity and unpleasantness of heat-pain tolerance and threshold in both settings where a dog is present. Moreover, we also hypothesized that the presence of a dog would strengthen the alliance between participant and the treatment provider. To examine possible effects of the presence of an animal on the therapeutic alliance, we assessed participants' perception of the study investigator in all pain assessments.

## Methods

### Design

We conducted a randomized controlled trial with four experimental conditions and healthy participants. In the pain assessment context, experimental pain was induced and assessed with a standardized experimental heat-pain paradigm, simulating a setting in which persons experience pain without treatment. In the pain therapy context, experimental pain was induced, assessed with a standardized experimental heat-pain paradigm, and, in addition, we employed an established expectation-induced placebo paradigm. In this context, we introduced placebo as therapeutic intervention for the experimentally induced pain to simulate a setting in which persons experience pain and get a treatment. A positive verbal suggestion was administered to induce expectation in relation to the placebo intervention. No positive verbal suggestion was administered in relation to the dog's presence to suppress possible expectation effects. Participants were randomly assigned to pain assessment (“pain”), pain assessment in the presence of a dog (“pain + dog”), pain assessment and a placebo intervention only (“pain + placebo”), or pain assessment and a placebo intervention in the presence of a dog (“pain + placebo + dog”).

The study protocol ensured the dog's welfare at any time. We conducted all dog sessions according to the guidelines of the International Association for Human-Animal Interaction Organizations (1).

The study was conducted between April 2019 and July 2019. The study protocols and the informed consent of the study were approved by the Ethics Committee of the Faculty of Psychology at the University of Basel, Switzerland.

### Participants

Through online advertisements, 284 participants were recruited for a study on pain perception at the University of Basel. The online advertisement did not contain any information about the possible presence of a dog to prevent attracting participants with an affinity for dogs. The online advertisement contained a link to a short questionnaire. Participants interested in participating had to complete this questionnaire first to check for eligibility and inclusion and exclusion criteria. Participants had to be (a) right-handed (32) and (b) 18 years or older to be included in the study. Exclusion criteria were (a) any acute or chronic disease as well as skin pathologies, (b) current medications or current psychological or psychiatric treatment, (c) pregnancy, (d) nursing, (e) current or regular drug consumption, (f) insufficient German language skills, (g) a fear of dogs, (h) dog-hair allergies, and (i) previous participation in studies using a heat-pain paradigm.

Of the total 284 screened participants, 201 met the inclusion criteria. All eligible participants received the study information, which contained the whole study procedure, aims, participants' rights, notification of the possible presence of a dog, and a selection of study appointments. After receiving all information about the study, a total of 159 participants were willing to participate in the study (a detailed overview of the enrollment can be found in the Supplementary Material, F1). Participants who were still willing to participate were asked to sign in for a study appointment. As soon as the scheduled *N* = 132 participants confirmed their study appointments, the remaining people were informed that there were no further appointments available. Participants attended one appointment that took about 70 min. The study compensation was CHF 80. Psychology students had the opportunity to obtain credit points for study.

Participants were blinded regarding the aims of our study and the placebo intervention. At the end of the study, all participants provided delayed informed consent, which debriefed them about the aims of the study. Participants were able to withdraw data from the study if they did not consent to participate anymore.

### Randomization

We used an adaptive randomization to apportion male participants over all four conditions because we expected more women than men to participate in the study. This approach automatically considered the previous gender allocation in the four conditions and influenced the probability of the next gender allocation. This ensured that gender was equally represented in all four conditions (“pain,” “pain + dog,” “pain + placebo,” “pain + placebo + dog,” each *N* = 33). The randomization was conducted with Microsoft® Excel for Mac, version 16.16.17. The first author entered participant's code and gender into the Excelfile which then automatically allocated participants to one of the four study conditions. Participants did not know in which condition they were until the treatment phase. The study investigators, however, were not blinded as they knew in which condition the participant was.

### Procedure

After guiding a participant into the room, the study investigator explained the study procedure to the participant and asked them to fill in the sociodemographic questionnaire, which took about 10 min. Then baseline measurements of heat-pain tolerance and threshold as well as subjective pain ratings were collected for each participant. This baseline procedure lasted 20 min.

After these baseline measurements, the treatment phase was conducted; it took a total of 15 min. Participants in the AAI conditions were introduced to the dog. They were deceived about the real reason for the dog's presence (to investigate the effect of the mere presence of a dog) so as to suppress possible expectation effects. Participants were informed that the dog had to be acquainted with the study procedure to be able to participate in a future study. They were told that the dog would rest quietly on a blanket and would not disturb the study procedure. To standardize the interaction between the participants and the dog, all participants were asked to greet and pet the dog as soon as it entered the room. We explained that it would be easier for the dog to relax on a blanket when allowed to greet the new person in the room. The duration of the interaction between the participant and the dog was kept to minimum, that is, under 1 min. During the greeting phase the study investigator also interacted with the dog, if the dog approached the investigator. After this greeting phase, the dog was asked to lie on its blanket, which was always next to the participant so that participants could still see the dog. Participants did not touch the dog during the further procedure. The study investigator also did not interact with the dog during the further procedure. The dog was a one-and-a-half-year-old female Golden Retriever used interacting with unfamiliar people. All conditions without a dog were carried out by three other female study investigators. All dog conditions were performed by the same female study investigator, who was the dog's owner. The reason for this was to ensure that the dog is not stressed. Leaving the dog in a setting with unfamiliar individuals without the dog's owner would have been inappropriate from an ethical standpoint. All study investigators were instructed to follow a study manual describing all the procedures and the instructions of the participants.

After this introduction, the study investigator applied an inert white cream on the participants in all four conditions. However, the rationale differed in the four conditions. Participants in the two placebo conditions (“pain + placebo” and “pain + placebo + dog”) were told: “You will receive a generic analgesic cream with the active ingredient lidocaine. Lidocaine is the main ingredient of the analgesic cream Stilex (a local anesthetic commonly used in Switzerland). The cream prevents and treats itchy and painful skin problems, such as light burns, sunburns, or insect bites. The efficacy of lidocaine has been evidenced in several high-quality studies.” Participants in the two pain-assessment conditions (“pain” and “pain + dog”) were told: “You will receive a cream (hand cream) to moisturize the skin. This allows accurate pain measurements.”

**Figure 1:**
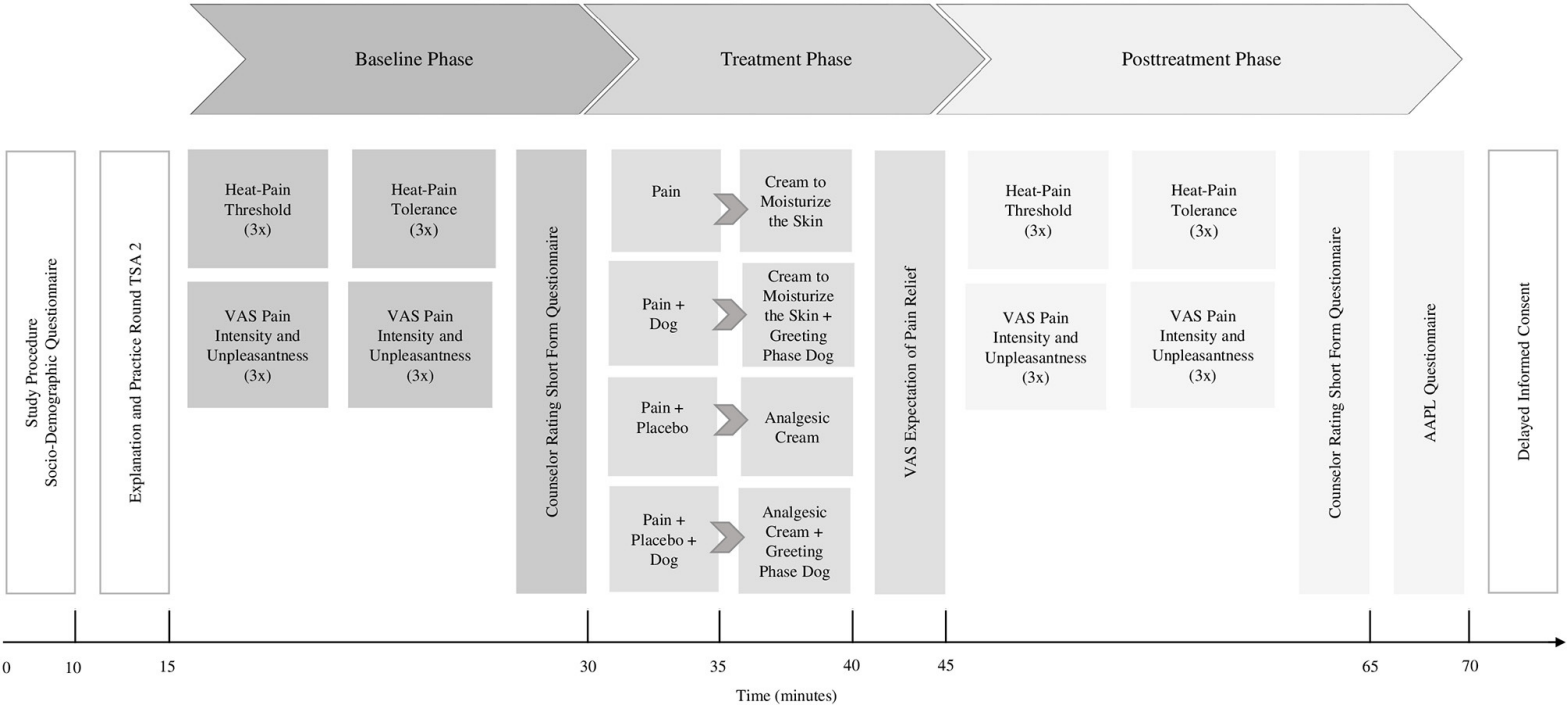
Timeline of the study procedure.

After the treatment phase, posttreatment heat-pain measurements and subjective ratings of pain intensity and unpleasantness were performed in an identical manner to the baseline assessments and lasted 20 min. At the end of the study, all participants provided delayed informed consent (see Figure 1 for the timeline of the study procedure).

## Measures

### Pain Ratings

We assessed heat-pain tolerance and heat-pain threshold following the design of previous trials (33–35). We defined posttreatment heat-pain tolerance as the primary outcome. Heat-pain tolerance is related to affective and motivational aspects (33, 36) and implies experiencing maximum discomfort, which results in greater subjective stress (33). In addition, it has been associated with pathological pain, as there is an inverse relationship between ischemic pain tolerance and the perceived severity of clinical pain (37). Posttreatment heat-pain threshold was defined as a secondary outcome. Both, the heat-pain threshold and heat-pain tolerance were determined using the Thermal Sensory Analyser (Medoc, Ramatishai, Israel; TSA 2). The heat-pain threshold was measured prior to heat-pain tolerance in order to minimize interference between the two outcomes (34, 35). The TSA 2 is a pain management system for qualitative assessment of pain and measures sensory thresholds such as heat-induced pain. The employed heat stimuli did not entail any significant danger and have already been used in previous studies in our lab (30, 34, 35, 38, 39). Participants were able stop the stimuli at any time during each experimental run.

The study investigator administered the heat stimuli to the right volar forearm of the participant using a 30 × 30 mm Peltier device (Medoc, Ramatishai, Israel; TSA 2). The thermode of the TSA 2 was fixed at two different locations (locations Y and X, determined using a positioning device). Location Y was placed one-third away from the elbow, while location X was placed two-thirds away from the elbow. Half of the participants were randomly assigned to start with location Y for the baseline heat-pain measurement and to switch then to location X for the posttreatment heat-pain measurement. The other half of the participants started with the opposite location, location X first for the baseline heat-pain measurement followed by location Y for the posttreatment measurement. The reason for moving the thermode was to avoid effects of sensitization or habituation (40).

Before starting with the actual heat-pain measurement, participants performed a practice round to experience how the heat stimuli work and how to handle the device including how to stop the heat stimuli. After this practice round, we started with the baseline measurements. We first assessed heat-pain threshold which was determined by the method of limits. Participants were instructed to press the button to determine the turning point from perceiving warmth to perceiving pain. The temperature was increased from the baseline (32°C) at a rate of 0.5°C/s. When participants indicated that the pain threshold had been reached, the device resumed from its baseline (32°C) with a rise of 0.5°C/s. This procedure was repeated three times in a row (35). The heat-pain threshold was defined as the average of the three measurements.

Afterward, heat-pain tolerance was determined using the method of limits. Participants were asked to stop the increasing heat stimulus at the moment they could not stand the heat any longer. The temperature increased from the baseline (32°C) at a rate of 0.5°C/s. As soon as participants indicated that their pain tolerance had been reached, the device resumed from its baseline (32°C) with a rise of 0.5°C/s. Again, this procedure was repeated three times in a row (35). To avoid physical injury, the pain tolerance measurement stopped at a temperature of 52°C (41). Heat-pain tolerance was defined as the average of the three measurements (42).

The secondary outcomes were the subjective pain-intensity rating of heat-pain tolerance, the subjective pain-intensity rating of the heat-pain threshold, the subjective unpleasantness rating of heat-pain tolerance, the subjective unpleasantness rating of the heat-pain threshold, and pain expectation.

Subjective pain-intensity and unpleasantness ratings of heat-pain tolerance and of the heat-pain threshold were measured with a visual analogue scale (VAS). The VAS ranged from 1 to 10 (1 = “not intense at all” or “not unpleasant at all”; 10 = “the most intense pain I have ever experienced” or “the most unpleasant pain I have ever experienced”). Participants were asked to evaluate subjective pain intensity and unpleasantness after each objective pain measurement. Subjective pain intensity and unpleasantness are assessed pain parameters in heat pain paradigm studies (43). Intensity refers to cognitive dimensions of pain, whereas unpleasantness refers to the affective dimension of pain (44).

After the treatment phase and before conducting the posttreatment heat-pain measurements, participants were asked to indicate on a VAS how intense they expect pain to be after the treatment phase. These expectation ratings were made on the same VAS (ranging from 1 to 10) as those for pain intensity and pain unpleasantness (35). Pain expectation was assessed to control if the expectation-induced placebo intervention was successful.

### Participants' Perception of the Study Investigator

Participants' perception of the study investigator was assessed with the Counselor Rating Form–Short Version (CRF-S) (45). The CRF-S is a 12-item questionnaire for measuring an individual's perception of the therapist on the following three subscales: *trustworthiness, expertness*, and *attractiveness*. The questionnaire contains items on a 7-point Likert scale, ranging from 1 (not very) to 7 (very). For this study, only the subscale *trustworthiness* was analyzed because it is most central to the therapeutic alliance. Studies indicate that patient trust in the physician is of particular importance in clinical practice (46–48). The subscale *trustworthiness* included the following four items: *honest, reliable, sincere and trustworthy*. The CRF-S was used twice in the study: first after the baseline assessments and second after the posttreatment assessments. Due to an online survey programming error the item *honest* of the subscale *trustworthiness* has not been collected within the first 31 participants. As the other tree items of the subscale *trustworthiness* were completed, this has been defined as item-level missingness (49). To treat these missing items, the mean across available items was taken, as recommended by Roth et al. (50).

### Demographic Variables

Before the study start, we assessed demographic variables (i.e., age, sex, nationality, family status, educational level, employment situation, and income) with the sociodemographic questionnaire.

### Dog Related Variables

The study investigator quantified the intensity of the contact between participant and dog during the greeting phase with a 5-stage Likert scale. The Likert scale ranged from 1 = “no contact at all” to 5 = “very high intensity of contact.” Further, we assessed the participants affinity for dogs at the end of the study with a short self-developed questionnaire. We used a 5-stage Likert scale, with 1 indicating that participants like dogs “not at all” and 5 indicating “very much.”

### Data Analysis

We estimated that a sample size of *N* = 128 with a power of 0.8, an alpha error of 5% and a beta error of 20% would be necessary to detect a medium size effect of *f* = 0.25 between the four conditions, as well as interaction between them (7). We decided to add *N* = 4 (one person in each condition) in case of dropouts during the study or data loss due to technical problems. We therefore included 132 participants.

The primary outcome (posttreatment heat-pain tolerance) was analyzed using linear models (analysis of covariance, ANCOVA) with the corresponding baseline outcome of heat pain tolerance as a covariate. We wanted to investigate how the dog affects pain perception in the two different contexts—pain assessment and pain therapy—by comparing “pain” with “pain + dog” and “pain + placebo” with “pain + placebo + dog.” We also run both models for the primary outcome twice, including gender and once including age (not pre-specified).

For the secondary outcomes (the posttreatment heat-pain threshold and the corresponding subjective pain-intensity and unpleasantness ratings of heat-pain tolerance and of the heat-pain threshold), we also conducted linear models (ANCOVAs) comparing “pain” with “pain + dog” and “pain + placebo” with “pain + placebo + dog.” In each model, the respective corresponding baseline outcomes were used as covariates.

With regard to the subjective expectation ratings, we conducted a linear model (analysis of variance, ANOVA) using the four treatment conditions (“pain,” “pain + dog,” “pain + placebo,” and “pain + placebo + dog”) as an independent between-subject factor.

To analyze the subscale *trustworthiness* of the CRF-S questionnaire, we conducted a linear model (analysis of covariance, ANCOVA) to investigate whether the presence of the dog affected the perception of the participants. Dog was used as an independent factor and the corresponding baseline outcome of the subscale *trustworthiness* was used as a covariate. In a second step, the same model was run with the four study investigators as a covariate. To control whether there was a difference between the four study investigators, another model was calculated including the study investigator as a factor.

The requirements for the analyses were tested using Levene's test to determine the variance homogeneity of the four conditions, the homogeneity of the regression slopes, and the normal distribution of the variables were tested using Shapiro-Wilk's test and quantile-quantile plot (Q-Q plot). All variables were normally distributed and all requirements were met. The prerequisites of ANCOVA were also met. There were no significant differences in baseline pain scores and in the CRF-S questionnaire between the four conditions. Further, there was a linear relationship between each covariate, in our case the corresponding baseline value, and the dependent variable, in our case the corresponding posttreatment value. We reported our outcomes according to the Consolidated Standards of Reporting Trials (CONSORT) guidelines that suggest using the estimate with the confidence interval. The mean difference (estimate) was used as effect size, the confidence interval was defined at 95% and the significance level was set at 0.05. All statistical analyses were carried out using R for Mac, version 1.4.1103.

## Results

### Sample Characteristics

All 132 participants were included in the analysis. Participants had a mean age of 26.2 (*SD* = 8.3). Eighty-eight participants were females, and 44 were males. Participants in the four conditions did not differ regarding age (pain: mean age = 26.58, SD = 10.03; pain + dog: mean age = 26, SD = 6.13; pain + placebo: mean age = 24.62, SD = 7.06; pain + placebo + dog: mean age = 27.39, SD = 9.38), gender, family status, educational level, or employment level (see Table 1). In addition, we also analyzed if there were differences between the conditions “pain” and “pain + dog” and the condition “pain + placebo” and “pain + placebo + dog” separately. No differences were found; detailed outcomes can be found in the (Supplementary Materials 1, 2). Moreover, we also analyzed potential differences between the conditions “pain + dog” and “pain + placebo + dog” regarding the intensity of interaction between the participants and the dog or regarding the participants' dog affinity. No differences were found; detailed results can be found in the (Supplementary Material 3).

**Table 1:** Sociodemographic characteristics of participants.

**Condition**	***N***	**Age mean (*SD*)**	***N* (%) female**	**Family status *N***	**Highest educational level *N* (%)**	**Employment level *N* (%)**
Pain	33	26.58 (10.03)	23 (69.69%)	Single: 32 Married: 0 Registered partnership: 0 Divorced: 0 Other: 1	Primary school: 0 Secondary school: 1 (3.03%) High school: 19 (57.57%) University: 13 (39.39%)	Full time: 3 (9.09%) Part time: 8 (24.24%) None or student: 22 (66.66%)
Pain + Dog	33	26 (6.13)	22 (66.66%)	Single: 31 Married: 1 Registered Partnership: 0 Divorced:0 Other: 1	Primary school: 0 Secondary school: 0 High school: 17 (51.52%) University: 16 (48.48%)	Full time: 5 (15.15%) Part time: 14 (42.42%) None or student: 14 (42.42%)
Pain + Placebo	33	24.64 (7.06)	23 (69.69%)	Single: 31 Married: 2 Registered partnership: 0 Divorced:0 Other: 0	Primary school: 0 Secondary school: 3 (9.09%) High school: 18 (54.55%) University: 12 (36.36%)	Full time: 2 (6.06%) Part time: 8 (24.24%) None or student: 23 (69.70%)
Pain + Placebo + Dog	33	27.39 (9.38)	20 (60.60%	Single: 29 Married: 3 Registered partnership: 0 Divorced: 0 Other: 1	Primary school: 0 Secondary school: 1 (3.03%) High school: 20 (60.60%) University: 12 (36.36%)	Full time: 8 (24.24%) Part time: 6 (18.18%) None or student: 19 (57.58%)

*SD, standard deviation*.

### Primary Outcome: Heat-Pain Tolerance

**Figure 2:**
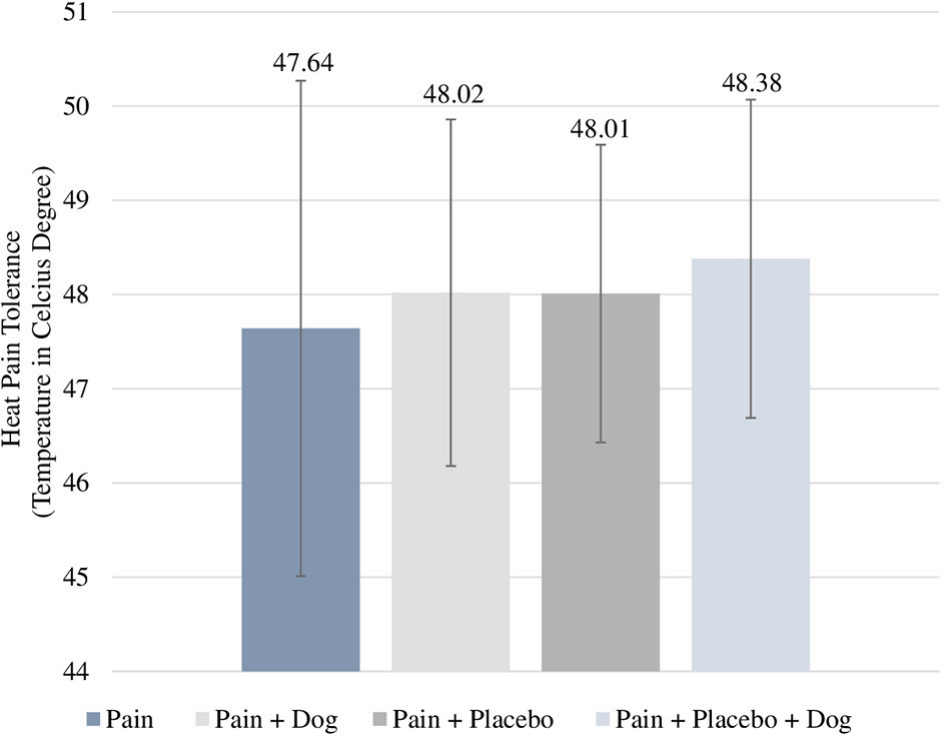
Posttreatment mean scores of heat-pain tolerance. For each condition, the respective mean and standard deviation are displayed.

We observed a mean posttreatment heat-pain tolerance of 47.64 in the “pain” condition which did not differ significantly from 48.02 in the “pain + dog” condition (difference = 0.04, CI = −0.66 to 0.74, *p* = 0.905). The posttreatment heat-pain tolerance mean value in the “pain + placebo” condition was 48.01 and did also not significantly differ from 48.38 in the “pain + placebo + dog” condition (difference = 0.43, CI = −0.02 to 0.88, *p* = 0.059) (see Table 2; Figure 2). Baseline heat-pain tolerance was associated with *p* < 0.001 in both models.

**Table 2:** Heat-pain tolerance and corresponding subjective intensity and unpleasantness ratings [mean, standard deviation (SD)].

		**Condition**			
		**Pain** **(*****N*** **=** **33)**	**Pain** **+** **Dog** **(*****N*** **=** **33)**	**Pain** **+** **Placebo** **(*****N*** **=** **33)**	**Pain** **+** **Placebo** **+** **Dog** **(*****N*** **=** **33)**
Baseline	Heat-pain tolerance (mean, SD)	48.06 (2.12)	48.41 (1.51)	48.29 (1.22)	48.22 (1.70)
	Subjective heat-pain intensity (mean, SD)	6.83 (1.52)	7.24 (1.45)	7.06 (1.43)	6.96 (1.45)
	Subjective heat-pain unpleasantness (mean, SD)	6.72 (1.73)	7.07 (1.30)	6.73 (1.85)	6.53 (1.79)
Posttreatment	Heat-pain tolerance (mean, SD)	47.64 (2.63)	48.02 (1.84)	48.01 (1.58)	48.38 (1.69)
	Subjective heat-pain intensity (mean, SD)	6.83 (1.49)	7.57 (1.36)	7.04 (1.75)	7.01 (1.66)
	Subjective heat-pain unpleasantness (mean, SD)	6.89 (1.87)	7.14 (1.41)	6.64 (2.12)	6.63 (1.91)

When including age in the model comparing the conditions “pain” and “pain + dog,” age has no effect on posttreatment heat-pain tolerance (difference = 0.58, CI = −0.03 to 0.05, *p* = 0.701) and the conditions “pain” and “pain + dog” did not differ regarding posttreatment heat-pain tolerance (difference = 0.05, CI = −0.66 to 0.75, *p* = 0.891). In the comparison “pain + placebo” with “pain + placebo + dog” there was an age effect (difference = −0.04, CI = −0.07 to 0.01, *p* = 0.002) and the conditions “pain + placebo” and “pain + placebo + dog” significantly differed (difference= 0.54, CI = 0.12–0.97, *p* = 0.013). Baseline heat-pain tolerance was associated with *p* < 0.001 in both models.

When including gender into the model no changes to the original model were found. Gender had no effect on posttreatment heat-pain tolerance when comparing the conditions “pain” and “pain + dog” (difference = −0.10, CI = −0.87 to 0.66, *p* = 0.785). There was no difference between “pain” and “pain + dog” in posttreatment heat-pain tolerance (difference = 0.04, CI = −0.66 to 0.75, *p* = 0.902). When comparing the conditions “pain + placebo” and “pain + placebo + dog,” we found no effect of gender (difference = 0.20, CI = −0.28 to 0.67, *p* = 0.407) and no group differences in posttreatment heat-pain tolerance (difference= 0.41, CI = −0.04 to 0.86, *p* = 0.073). Baseline heat-pain tolerance was associated with *p* < 0.001 in both models.

### Secondary Outcomes

#### The Heat-Pain Threshold, Subjective Pain Intensity and Unpleasantness of Heat-Pain Tolerance, Subjective Pain Intensity and Unpleasantness of the Heat-Pain Threshold

There was no significant effect of the dog on the posttreatment heat-pain threshold; detailed outcomes can be found in the (Supplementary Material 4, T1).

**Figure 3:**
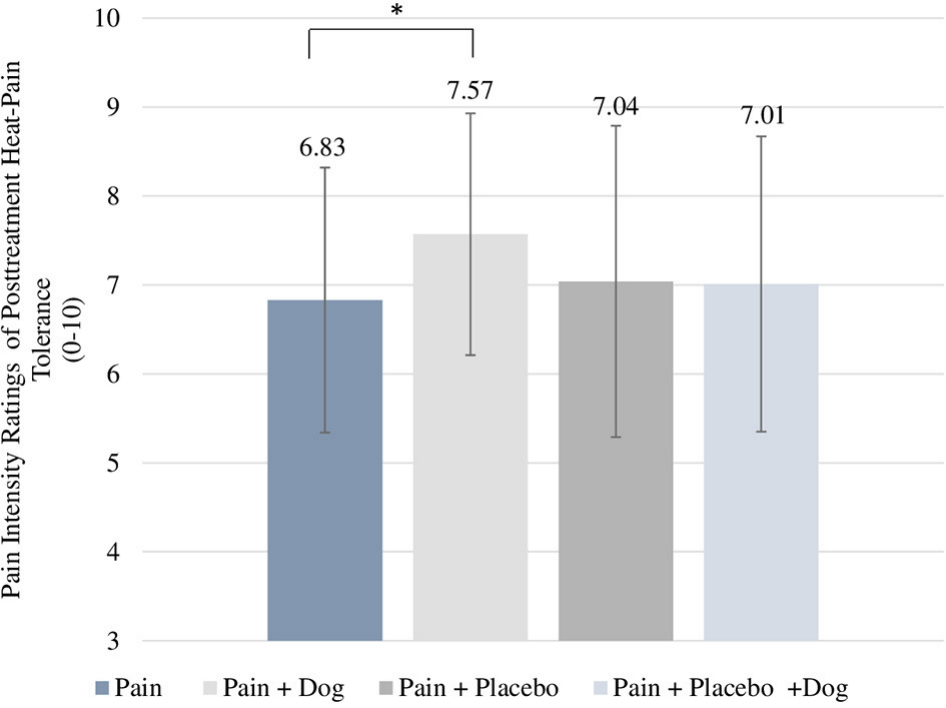
Posttreatment scores of subjective intensity ratings for heat-pain tolerance. For each condition, the respective mean and standard deviation are displayed. *p-value < 0.05.

With regard to the subjective intensity rating of heat-pain tolerance the “pain” had a mean value of 6.83 which was significantly lower than 7.57 in the “pain + dog” condition. This indicates that participants in the “pain + dog” condition experienced higher pain intensity of heat-pain tolerance compared to participants in the condition “pain” (difference = 0.40, CI = 0.02–0.79, *p* = 0.041) (see Table 2; Figure 3). Further, “pain + placebo” had a mean value of 7.04 which did not significantly differ from 7.01 in “pain + placebo + dog” condition (difference = 0.07, CI = −0.38 to 0.52, *p* = 0.754) (see Table 2). Baseline subjective ratings of pain intensity of heat-pain tolerance was associated with *p* < 0.001 in both models.

With regard to the subjective unpleasantness rating of heat-pain tolerance, the dog had no effect. There was no significant difference between mean value of 6.89 in the “pain” condition compared to the mean value of 7.14 in “pain + dog” condition (difference = −0.03, C = −0.59 to 0.53, *p* = 0.913) or between the mean value of 6.64 in the “pain + placebo” condition and the mean value of 6.63 in the “pain + placebo + dog” condition (difference = 0.19, CI = −0.29 to 0.67 *p* = 0.44). Baseline subjective ratings of pain unpleasantness of heat-pain tolerance was associated with *p* < 0.001 in both models.

With regard to the subjective intensity and unpleasantness rating of the heat-pain threshold there were no differences among the conditions; detailed outcomes can be found in the (Supplementary Material 5, T1).

#### Expectation of Pain Reduction

We found no differences between the four conditions regarding their expectation of pain reduction after treatment (difference = −0.17, CI = −0.45 to 0.11, *p* = 0.241). Separate analysis of the conditions also showed no difference regarding pain expectation between the conditions “pain” with a mean value of 5.41 and the mean value of 5.36 in the “pain + dog” condition (difference = 0.04, CI = −0.88 to 0.97, *p* = 0.927) or the conditions “pain + placebo” with a mean value of 4.81 and the mean value of 5.03 in the “pain + placebo + dog” condition (difference = −0.22, CI = −1.09 to 0.66, *p* = 0.620).

#### Perception of the Study Investigator

There was no significant effect of the dog on the trustworthiness of the study investigators (see Table 3). The ratings of trustworthiness of the study investigators in the condition “pain” with a mean value of 25.94 did not differ from the mean value of 26.76 in the condition “pain + dog” (difference = 0.10, CI = −0.67 to 0.87, *p* = 0.796). The ratings of trustworthiness of the study investigators in the condition “pain + placebo” with 25.52 did not differ from 26.48 in the condition “pain + placebo + dog” (difference = 0.11, CI = −0.43 to 0.64, *p* = 0.695). Baseline trustworthiness ratings of the study investigators was associated with *p* < 0.001 in both models. When we controlled for study investigator, there was still no significant difference in the subscale *trustworthiness* of the study investigators between the four different investigators comparing the conditions “pain” with “pain + dog” (difference = −0.06, CI = −1.46 to 1.35, *p* = 0.936) or between the conditions “pain + placebo” and “pain + placebo + dog” (difference = 0.26, CI = −0.74 to 1.27, *p* = 0.601). Baseline trustworthiness ratings of the study investigators was associated with *p* < 0.001 in both models.

**Table 3:** Counselor Rating Short Form Questionnaire (CRF-S): Subscale Trustworthiness [mean, standard deviation (SD)].

		**Condition**			
		**Pain** **(*N = 33)***	**Pain + Dog** **(*N* = 33)**	**Pain + Placebo** **(*N* = 33)**	**Pain + Placebo + Dog** **(*N* = 33)**
Trustworthiness	Baseline (mean, SD)	25.42 (3.25)	26.58 (2.18)	25.70 (3.10)	26.58 (2.19)
	Posttreatment (mean, SD)	25.94 (2.90)	26.76 (2.28)	25.52 (3.26)	26.48 (2.36)

The results of the subscales attractiveness and expertness can be found in the (Supplementary Materials 6).

## Discussion

AAIs have been shown to be effective in the treatment of pain, but the mechanisms of this analgesia have not yet been elucidated. This study investigated whether the analgesic effects of AAI could be mediated by providing direct social support through the presence of a dog or by strengthening the alliance between the patient and the treatment provider. We tested these hypotheses with established paradigms for pain assessment and pain therapy, i.e., expectancy-induced placebo analgesia.

The results of our randomized controlled trial show that participants heat-pain tolerance did not increase in both pain assessment and pain therapy when a dog was present. Instead, subjective measures show that participants experienced heat-pain tolerance to be more intense when the dog was present compared to when no dog was present in the pain assessment condition where no treatment was offered. Further, participants did not perceive the study investigator to be more trustworthy when a dog was present compared to when no dog was not present. These results contradict our assumption that the analgesic effects of AAI could be mediated by providing direct social support or by strengthening the alliance between the participant and the treatment provider.

These findings also contradict previous observations of analgesia in the presence of a dog in a clinical setting (11–14, 51) but are in line with studies that found no effect of AAI in pain (16, 17). Moreover, we did not only find no analgesic effect of the dog but instead a negative effect in the subjective pain intensity of heat-pain tolerance. To our knowledge, this is the first study that found a negative effect of AAI on pain. There are several possible explanations for this discrepancy between our findings and previous studies.

These contradict results could be a consequence of differences in the study setting as we employed an experimentally induced acute pain paradigm in healthy participants, whereas previous studies reported pain reduction in patients in the presence of a dog compared to patients without a dog present in a clinical setting (11–14, 51).

Further, it is possible that for AAI to be effective, the animal (in our case, a dog) needs to be actively involved in giving social support to modulate pain, for example, through direct physical contact or a clear attentional focus of the animal toward the human. This would be in line with a previous meta-analysis on the analgesic effects of human social support suggesting that the mere presence of another person is not sufficient to affect pain perception and experience and that social support needs to be expressed clearly in order to reduce pain, for example, through verbal communication or holding hands (19). It is therefore possible that a dog also needs to be actively involved in the therapeutic process in order to modulate pain. Accordingly, in previous studies that have suggested that dogs affect patients' pain perception, patients typically interacted with the dogs for 10–20 min (11–13, 51). This would also be in line with previous studies showing that physical contact between a human and an animal is important to stimulate biological reactions in humans (52–54). Notably, these effects might not only rely on physical contact since both physically interacting with and just seeing a dog increases oxytocin level in humans (23). Based on these findings as well as on our results, we assume that the mere presence of a dog is not sufficient to affect pain perception and that at least a longer interaction phase and some form of contact between the human and the animal might be needed. Further, it can be important whether the person knows or owns the animal. Support for this assumption comes from a study that examined the effect of the presence of friends, spouses and pet on cardiovascular responses to psychological and physical stress. The authors showed that pet owners perceive their pets as an important, supportive part of their lives, and significant cardiovascular and behavioral benefits are associated with this perception (55, 56). In our study, participants did not know the dog. So, it is possible that a relationship needs to exists between human and animal for the presence of an animal to have a positive effect. Future studies should investigate if the relationship to the animal mediates a possible analgesic effect.

Another explanation is based on findings from placebo and psychotherapy research. Studies have shown that a treatment rationale is an important prerequisite for a treatment response (30, 35, 39). In our experiment, we used a deceptive rationale for the dog's presence, and we intentionally avoided a therapeutic narrative for the dog. However, research has indicated that interventions evoking expectations of pain reduction—either by verbal suggestion, conditioning, or imagery techniques—are likely to contribute to improving the effectiveness of standard analgesic treatments in clinical practice (57). Further, depending on the information given in verbal suggestions, the verbal suggestion of an analgesic treatment can lead to different magnitudes of analgesia (58–61). For example, a positive expectation leads to significant pain reduction, whereas a verbal suggestion inducing negative expectations can even block a painkiller's analgesic effect. This leads to the assumption that positive and negative expectations can have an impact on the outcome of an intervention (62). Hence, it is possible that we did not find an analgesic effect of the dog because participants lacked the grounds to incorporate the dog in their treatment expectations. Moreover, it is even possible that the dog was then perceived as a negative distraction. This would also explain why participants in the “pain + dog” condition experienced greater pain intensity compared to participants in the “pain” condition. This would also mean that the effect of AAI on pain reduction cannot be explained solely by the animal but is rather influenced by contextual factors, such as expectation.

Further, it could be that by not providing any information regarding the presence of a dog during the recruitment process, we might have attracted participants with no specific attitudes toward dogs. In our study dog affinity was only collected to check that groups did not differ regarding their dog affinity. However, it has been suggested that individuals with an affinity for animals may be more likely to benefit from their presence (14). It is possible that people with an affinity for dogs would more strongly benefit from a dog's presence. Thus, not limiting the study to people with an affinity for dogs could have led to a smaller effect of the dog's presence on pain perception and experience.

Last, the presence of a dog did not positively affect how participants perceived the study investigator. These results do not support findings of previous studies suggesting that the presence of an animal positively influences how we perceive others (25, 63). In both studies, participants perceived psychotherapists in images or videos with an animal present to be more attractive, and in a study by Schneider et al. (63), participants perceived the same psychotherapists as more trustworthy when an animal was present. However, our results are in line with the study by (26), who also found that the presence of a dog had no effect on participants' perception. A plausible explanation for the difference in results between, on the one hand, previous studies supporting a positive effect of animals on our perception (25, 63) and, on the other, our study and Goldmann et al.'s study is the study setting. In our study and in Goldmann's study, the effect of the presence of a dog on participants' perception was investigated *in vivo*. In both studies, there was direct interaction between the participant and the study leader, whereas in the previous studies the participants had to judge an image or video of a person with or without an animal and the participants did not interact with an animal or study leader. It is therefore possible that through this direct interaction between participant and study investigator, the dog was not the focus of participants and had no effect on their perception of the study investigator (26). However, since the dog conditions were only performed by one study investigator, these results must be interpreted with caution. With our design, it is difficult to compare the study investigator that worked with the dog with the other three study investigators.

Overall, the results of this study are not only interesting for research on AAIs but also for placebo research, especially from a methodological perspective. In this study, we used a placebo as an intervention paradigm to examine whether the presence of a dog could amplify the placebo effect. The placebo was thus not used as a control intervention to eliminate specific factors as is usually the case. Using a placebo as an intervention paradigm has been implemented in a few previous studies, for example, in those by (30, 31) investigated the effect of the patient–practitioner relationship on patients with irritable-bowel syndrome using a placebo acupuncture intervention; they suggested that an enhanced relationship with a practitioner is the most robust component in therapy. Further, Gaab et al. (30) examined the impact of expectation and relationships in healthy participants using a placebo intervention consisting of animated videos. The authors showed that placebos with a psychological treatment rationale are effective when provided in a trustworthy, friendly, and empathic relationship. In our study, we used the presence of a dog to examine whether the presence of a dog could amplify the placebo effect and found that the mere presence of a dog has no impact on the placebo effect.

However, it should also be emphasized that in this study, we did not succeed in inducing placebo effects. This finding contradicts results from previous studies (34, 35). A possible explanation for the lack of placebo effect might be that in this study, the expectation induction was not successful. As known from previous research treatment response expectation is generally seen as the main contributor to placebo-induced analgesia (64–66). Hence, we may not have been able to produce placebo effects since participants had no expectation of pain relief. Another possible explanation might be that the dog and not the placebo was the focus in our study. We used a placebo as an intervention paradigm and not to study placebo effects like in previous studies. As a result, it is possible that the study investigators did not have a placebo allegiance in this study. As known from psychotherapy research there exists a robust relationship between researcher allegiance and outcome (67). Hence, a potential missing placebo allegiance could lead to a lower expectation of pain reduction among participants and explain the lack of placebo effect in this study.

The findings of this study have to be seen in light of some limitations. Our sample consisted of young and healthy people who were not suffering from acute or chronic pain. While valuable evidence can be provided from studies in healthy participants, it is important to stress that short-term experimentally induced or acute pain in healthy participants differs from chronic pain in patients (68). Hence, our results only provide information about how the presence of a dog affects experimentally induced acute pain of healthy participants. Therefore, our results need to be treated with caution in the context of acute or chronic pain. Future studies should apply this design also with patients with pain disorders or patients experiencing acute pain in clinical settings. Further, the dog conditions were performed by the same person, while the other interventions were performed by different people. The results of the CRF-S questionnaire showed, however, that even when controlling for the investigator, there was no significant difference in how participants rated the study investigators. Finding no difference can lead to the assumption that all four investigators performed the intervention in the same standardized manner according to the manual. However, even though this analysis made us assume that all our study investigators performed the conditions in the same manner we need to highlight that with our design, it is not possible to distinguish between the effects of the dog and the study investigator. Future studies should make sure that the study investigators carry out both conditions with and without an animal present to entangle the effects of the animal and the effects of the study investigator. Further, participants had only limited contact with the dog since the aim of this study was to investigate whether the mere presence of a dog had an analgesic effect.

Last but not least, the intensity as well as dog affinity were collected in this study, but only to roughly investigate if the dog groups differ regarding the intensity of contact and their dog affinity. It would have been interesting to investigate whether dog affinity and intensity of the contact between the participants and the dog mediates the effect. We therefore suggest that future studies should specifically address the affinity of participants for animals in general as well as for the animal that is presented.

Considering the findings and limitations of this current study, future studies are warranted that would investigate whether animals need to be integrated in the treatment rationale in order to have effects on pain. Further, it is important to examine whether physical contact with a dog is needed for an analgesic effect or not and whether affinity toward dogs mediates this effect.

In conclusion, our results indicate that the mere presence of a dog does not contribute to pain reduction and that the previously reported analgesic effects of AAI is not replicated in our study. The presence of a dog did not seem to provide social support or had an effect on the alliance between the participants and the treatment provider. We assume that the animal might need to be an integrated and plausible part of the treatment rationale so that participants are able to form a treatment-response expectation toward AAI.

## Data Availability Statement

The raw data supporting the conclusions of this article will be made available by the authors, without undue reservation.

## Ethics Statement

The studies involving human participants were reviewed and approved by Ethics Committee of the Faculty of Psychology at the University of Basel, Switzerland. The patients/participants provided their written informed consent to participate in this study.

## Author Contributions

JG had the idea for the study. CW, KH, JG, and CL designed the study. CW contributed to acquiring the data. CW and CL carried out the analysis. CW, KH, JG, and CL wrote the manuscript, which was revised by all authors. All authors contributed to the article and approved the submitted version.

## Funding

KH received support from an Ambizione grant from the Swiss National Science Foundation (grant PZ00P1_174082). CL received funding from the Swiss National Science Foundation (grant P400PS_180730).

## Conflict of Interest

The authors declare that the research was conducted in the absence of any commercial or financial relationships that could be construed as a potential conflict of interest.

## Publisher's Note

All claims expressed in this article are solely those of the authors and do not necessarily represent those of their affiliated organizations, or those of the publisher, the editors and the reviewers. Any product that may be evaluated in this article, or claim that may be made by its manufacturer, is not guaranteed or endorsed by the publisher.
