# Supplementary material for: Lack of Effects of the Presence of a Dog on Pain Perception in Healthy Participants—A Randomized Controlled Trial
Source: Front Pain Res (Lausanne). 2021 Nov 5;2:714469. doi: 10.3389/fpain.2021.714469 (PMC8915708; doi:10.3389/fpain.2021.714469)
Supplement: Supplementary file 1 [file Data_Sheet_1.docx]

**SUPPLEMENTARY MATERIALS**

**Table of Contents**

S1: Results: Sample characteristics "Pain" vs. "Pain + Dog"

S2: Results: Sample characteristics "Pain + Placebo" vs. "Pain + Placebo + Dog"

S3: Results: Intensity Interaction with the Dog and Dog Affinity in "Pain + Dog" vs. "Pain + Placebo + Dog"

S4: Results: Heat-Pain Threshold

S5: Results: Subjective Ratings for Heat-Pain Threshold

S6: Results: Perception of the Study Investigator (Counselor Short Form Questionnaire)

ST1: Heat Pain Tolerance and Corresponding Intensity and Unpleasantness Ratings

SF1: Flow Chart

**S1: Results: Sample characteristics "Pain" vs. "Pain + Dog"**

|  | **Difference** | **95% confidence interval** | **p-value** |
| --- | --- | --- | --- |
| Age | 0.58 | -3.5 – 4.68 | 0.779 |
| Gender | -0.03 | -0.26 – 2.0 | 0.795 |
| Family status | -0.03 | -0.46 – 0.40 | 0.888 |
| Education level | -0.12 | -0.72 – 0.47 | 0.685 |
| Employment level | 0.33 | 0.04 – 0.71 | 0.078 |

**S2: Results: Sample characteristics "Pain + Placebo" vs. "Placebo + Dog"**

|  | | | |
| --- | --- | --- | --- |
|  | **Difference** | **95% confidence interval** | **p-value** |
| Age | -2.76 | -6.84 – 1.33 | 0.182 |
| Gender | -0.09 | -0.33 – 0.15 | 0.446 |
| Family status | -0.18 | -0.51 – 0.15 | 0.271 |
| Education level | -0.39 | -0.99 – 0.20 | 0.192 |
| Employment level | 0.33 | -0.06 – 0.73 | 0.094 |

**S3: Results: Intensity Interaction with the Dog and Dog Affinity in "Pain + Dog" vs. "Pain + Placebo + Dog"**

|  | | | |
| --- | --- | --- | --- |
|  | **Difference** | **95% confidence interval** | **p-value** |
| Interaction contact | 0.09 | -0.67 –0.49 | 0.755 |
| Dog affinity | -0.28 | -0.14 – 0.69 | 0.193 |

**S4: Results: Heat-Pain Threshold**

We observed a man posttreatment heat-pain threshold of 42.95 in the "pain" condition which did not differ significantly from 43.15 in the "pain + dog" condition (difference = 0.17, CI = -1.0 – 1.35, p = 0.772). The posttreatment heat-pain threshold mean value in the "pain + placebo" condition was 42.47 which did also not differ significantly from 43.61 in the "pain + placebo + dog" condition (difference = 0.93, CI = -0.05 – 1.90, p = 0.061). Baseline heat-pain threshold was associated with p < .001 in both models.

**S5: Results: Subjective Ratings for Heat-Pain Threshold**

Analysis showed no dog effect in pain intensity of heat-pain threshold between the condition’s "pain" and "pain + dog" (difference = 0.13, CI = -0.31– 0.57, p = 0.556) or between "pain + placebo only" and "placebo + dog" (difference = 0.30, CI = -0.06 – 0.67, p = 0.105). Baseline subjective ratings for pain intensity of heat-pain threshold was associated with p < .001 in both models. Further, there were also no significant differences in pain unpleasantness of heat-pain threshold between the condition’s "pain" and "pain + dog" (difference = 0.12, CI = -0.39 – 0.62, p = 0.643) or between "pain + placebo" and "pain + placebo + dog" (difference = 0.20, CI = -0.16 – 0.56, p = 0.267). Baseline subjective ratings for pain unpleasantness of heat-pain threshold was associated with p < .001 in both models.

**S6: Results: Perception of the Study Investigator (Counselor Short Form Questionnaire)**

There was no significant dog effect on the subscale’s *attractiveness* and *expertness.* The ratings of *attractiveness* of the study investigators with 25.53 in the "pain" condition did not differ from the rating of 25.39 in the "pain + dog" condition (difference = 0.46, CI = -0.19 –1.11, p = 0.160) or between the ratings of 25.64 in the "pain + placebo" condition and the ratings of 26.06 in the "pain + placebo + dog" condition (difference = 0.12, CI = -0.39 – 0.63, p = 0.630). Baseline ratings of *attractiveness* was associated with p < .001 in both models. Further, no significant differences were found in the ratings of *expertness* of the study investigators between 25.09 in the "pain only" and 25.82 in the "pain + dog condition (difference = 0.29, CI = -0.38 – 0.97, p = 0.393) or between the ratings of 24.33 in the "pain + placebo" condition and the ratings of 25.58 in the "pain + placebo + dog" condition (difference= -0.36, CI = -1.06 –0.33, p = 0.295). Baseline ratings of *expertness* was associated with p < .001 in both models.

**ST1: Heat-Pain Threshold and Corresponding Subjective Intensity and Unpleasantness Ratings**

*Heat-pain threshold and corresponding subjective intensity and unpleasantness ratings (mean, standard deviation [SD])*

|  |  | **Condition** | | | |
| --- | --- | --- | --- | --- | --- |
|  |  | **Pain**  (N = 33) | **Pain + Dog**  (N = 33) | **Pain + Placebo**  (N = 33) | **Pain + Placebo + Dog**  (N = 33) |
| **Baseline** | **Heat-pain threshold**  (mean, SD) | 43.86 (2.95) | 43.89 (2.77) | 43.77 (2.82) | 44.05 (2.68) |
|  | **Subjective heat-pain intensity**  (mean, SD) | 4.46 (1.77) | 4.10 (1.92) | 4.34 (2.13) | 3.99 (1.84) |
|  | **Subjective heat-pain unpleasantness**  (mean, SD) | 4.12 (1.77) | 3.90 (2.06) | 3.56 (1.86) | 3.60 (1.84) |
| **Posttreatment** | **Heat-pain threshold**  **(**mean, SD) | 42.95 (3.07) | 43.15 (3.42) | 42.47 (2.72) | 48.38 (3.00) |
|  | **Subjective heat-pain intensity**  (mean, SD*)* | 3.94 (1.62) | 3.77 (1.90) | 3.60 (1.87) | 3.57 (1.88) |
|  | **Subjective heat-pain unpleasantness**  (mean, SD) | 3.52 (1.55) | 3.4 (1.88) | 3.04 (1.57) | 3.25 (1.94) |

**SF1: Flow Chart**

## Enrollment

Analysed (n=33)

Analysed (n=33)

## Analysis

Allocated to "pain + placebo + dog"

(n= 33)

- Received allocated intervention (n= 33)

Allocated to "pain + placebo"

(n= 33)

- Received allocated intervention (n= 33)

Allocated to "pain" (n= 33)

- Received allocated intervention (n= 33)

Allocated to "pain + dog" (n= 33)

- Received allocated intervention (n= 33)

Randomized (n= 132)

Excluded (n= 152)

- Not meeting inclusion criteria (n= 83)
- Declined to participate (n=42)
- Other reasons (n= 27)

Assessed for eligibility (n= 284)

## Allocation

Analysed (n=33)

Analysed (n=33)
